# The reporting of study and population characteristics in degenerative cervical myelopathy: A systematic review

**DOI:** 10.1371/journal.pone.0172564

**Published:** 2017-03-01

**Authors:** Benjamin M. Davies, M. McHugh, A. Elgheriani, Angelos G. Kolias, Lindsay Tetreault, Peter J. A. Hutchinson, Michael G. Fehlings, Mark R. N. Kotter

**Affiliations:** 1 Division of Neurosurgery, Department of Clinical Neurosciences, University of Cambridge, Cambridge, United Kingdom; 2 Division of Neurosurgery and Spine Program Toronto Western Hospital, University Health Network & University of Toronto, Toronto, Canada; 3 WT MRC Cambridge Stem Cell Institute, Anne McLaren Laboratory, University of Cambridge, Cambridge, United Kingdom; 4 John van Geest Brain Repair Centre, University of Cambridge, Cambridge, United Kingdom; Universita degli Studi di Palermo, ITALY

## Abstract

**Object:**

Degenerative cervical myelopathy [DCM] is a disabling and increasingly prevalent condition. Variable reporting in interventional trials of study design and sample characteristics limits the interpretation of pooled outcomes. This is pertinent in DCM where baseline characteristics are known to influence outcome. The present study aims to assess the reporting of the study design and baseline characteristics in DCM as the premise for the development of a standardised reporting set.

**Methods:**

A systematic review of MEDLINE and EMBASE databases, registered with PROSPERO (CRD42015025497) was conducted in accordance with PRISMA guidelines. Full text articles in English, with >50 patients (prospective) or >200 patients (retrospective), reporting outcomes of DCM were deemed to be eligible.

**Results:**

A total of 108 studies involving 23,876 patients, conducted world-wide, were identified. 33 (31%) specified a clear primary objective. Study populations often included radiculopathy (51, 47%) but excluded patients who had undergone previous surgery (42, 39%). Diagnositic criteria for myelopathy were often uncertain; MRI assessment was specified in only 67 (62%) of studies. Patient comorbidities were referenced by 37 (34%) studies. Symptom duration was reported by 46 (43%) studies. Multivariate analysis was used to control for baseline characteristics in 33 (31%) of studies.

**Conclusions:**

The reporting of study design and sample characteristics is variable. The development of a consensus minimum dataset for (CODE-DCM) will facilitate future research synthesis in the future.

## Introduction

Chronic compression of the cervical spinal cord due to degenerative processes, including disc herniation, spondylosis, and ligament hypertrophy or ossification, has been collectively referred to as degenerative cervical myelopathy [DCM]. [1] Disability ranges from mild pain to severe sensorimotor deficits including quadriplegia. DCM is estimated to be the most common spinal cord disorder, and is expected to have an increasing incidence with the aging population in the industrial world [2].

Presently, surgical decompression is the mainstay of treatment, although the type and timing of surgery remains controversial. Defining optimal treatment strategies has been challenging due to difficulties in research synthesis and the heterogeneous reporting of outcome variables [3]. This is a recognized problem in many fields of healthcare and has led to the establishment of consensus-based, core outcome sets [4].

Effective pooled analysis and its accurate interpretation requires common outcome measures as well as an understanding of important study characteristics. This is particularly pertinent in DCM, where a number of baseline factors have been found to influence outcome [5,6]. Much like outcomes, these study components are often heterogeneous and not consistently reported. Pioneered by organizations such as the National Institute of Neurological Disorders [NIND], this has led to an extension of standardization from outcomes, to other study characteristics [7]. The nomenclature for this is inconsistent and includes ‘common data elements’ or ‘minimum reporting sets’.

Various methods have been proposed for the development of a minimum reporting set. One method is to map existing reporting practice by performing a systematic review of the literature. This information is then used to inform a DELPHI consensus process, that includes relevant stakeholders such as clinicians, academics, allied care professionals, patients and care givers. Organisations such as COMET [Core outcome measures in effectiveness trials] have been setup to facilitate this process [8].

The benefit of collaborative study in DCM is recognized. For example, the systematic and standardized approach of the AOSpine network has provided unique prospective datasets for advancing our understanding of DCM [9–12]. These have the potential to accelerate the development of optimal treatment for DCM, especially if future studies are designed on common grounds, and supported by a minimum reporting set.

Our objectives therefore were to describe the reporting of baseline characteristics in studies of DCM in order to inform a subsequent consensus process. This study complements and extends existing work on ancillary outcome measures in DCM and is referred to as CODE-DCM [Core outcomes and data elements in degenerative cervical myelopathy] [13,14].

## Method

A systematic review was conducted in accordance with the PRISMA guidelines (S1 Table) and registered with the PROSPERO (CRD42015025497) prospective register of systematic reviews. MEDLINE [Ovid] and Embase [Ovid] databases were searched on the 12^th^ August 2015 using the search strategy [“Cervical”] AND [“Myelopathy”] for articles focused on myelopathy secondary to chronic compression of the spinal cord. The search was conducted using the OVID Basic Search function. Related search terms were not included. Animal studies, case reports and letters/editorials were excluded.

Titles and abstracts were screened for relevance. Full text articles were subsequently screened for eligibility according to the following criteria:

- English, full text
- Prospective study with >50 patients or retrospective study with >200 patients
- Assessment of clinical outcomes in response to a treatment strategy (conservative or interventional)
- Articles published since 1^st^ January 1995

Articles were screened by two authors [BMD, AE] and data were extracted independently by two authors [BMD, MM] using a piloted proforma (S2 and S3 Tables). Discrepancies were settled by discussion and mutual agreement. A retrospective review of prospectively collected data was considered a prospective study.

Descriptive statistics were used to report frequency and proportion of measured data elements. Statistical comparisons were made using the Chi-Squared test, with significance set at p = 0.05.

## Results

**Fig 1:**
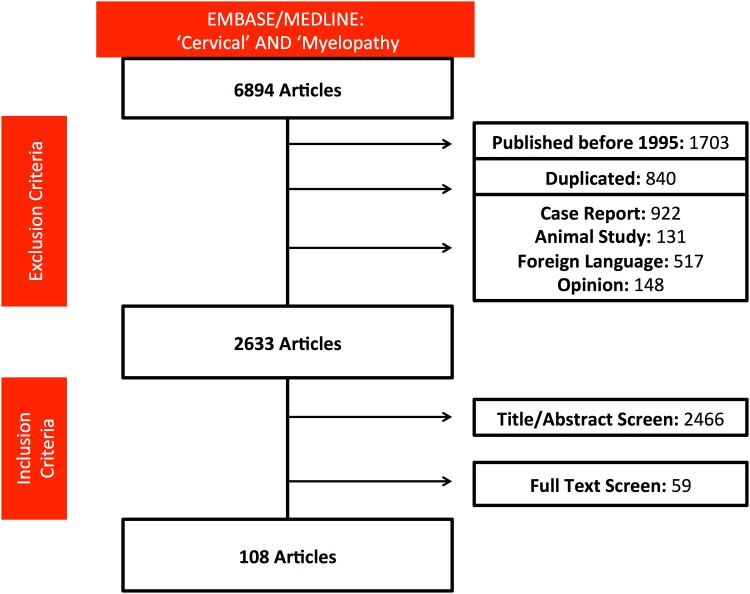
PRISMA flow diagram of search strategy.

The search strategy returned 6894 articles. Following application of inclusion and exclusion criteria, 108 articles were considered [Fig 1]. There were 91 prospective studies and 17 randomised controlled trials [RCT]. Further details about the shortlisted studies are available in our previous publication [3]

### Study design and patient selection

Of the 108 studies, 53 (49%) recorded whether ethical approval was obtained, including one study which cited that it was not required. Overall study objectives were outlined in 103 (95%) of studies; however, they were rarely specific. Thirty-three (31%) clearly specified a primary objective, including the timing of outcome assessment and 36 (33%) included secondary objectives. The investigation time period was specified in 93 (86%) studies and measured outcomes were defined in 96 (89%).

Clear inclusion and exclusion criteria were described in 98 (91%) and 75 (69%) studies, respectively. Patients who had previous cervical surgery were excluded by 42 (39%) studies. The diagnostic criteria for myelopathy were often unclear, with MRI assessment specified in only 67 (62%) studies. Neurophysiology for diagnosis was reported in two studies. Many studies included patients with myelopathy and radiculopathy; only 57 (53%) studies considered myelopathy only patients. The frequency of causative pathology (e.g. Disc herniation, OPLL) was specified in 87 (81%) of studies.

### Patient characteristics

Most articles reported disease severity (97, 90%) using one or more functional assessment tools, including the Japanese Orthopaedic Association assessment [JOA] (50, 46%), Nurick score (25, 23%), modified JOA (20, 19%) or the Oswestry Neck Disability Index [NDI] scales (20, 19%).

Imaging, distinct to that required for assessment of radiological outcomes or diagnosis, was reported by 59 (55%) studies. Typically this was MRI (58, 54%).

Imaging was used to report the disease level (46, 43%), number of treated levels (72, 67%) or putative prognostic factors (28, 26%) such as cord signal change (22, 20%) or cord compression measures (18, 17%).

Patient age (107, 99%) and gender (105, 97%) were typically recorded in studies. Race was recorded by 4 (4%) studies. General health status was referenced by 37 (34%) studies, typically by reporting on study specific subcategories such as BMI (13, 12%), smoking status (23, 21%), diabetes (9, 8%) or atherosclerotic disease (3, 3%). Only 9 studies used a recognized grading system: ASA [American Society of Anesthesiologists] (4, 4%), CCI [Charlson Comorbidity Index] (3, 3%) or CIRS [Cumulative Illness Rating Scale] (1, 1%). Other, less frequently reported patient information included employment status, workers compensation, mental health and medication burden. Symptom duration was reported by 46 (43%) studies. Multivariate analysis was used to control for baseline characteristics when evaluating outcomes, in 33 (31%) of studies.

### Operative and post-operative course

The technical details of the intervention were detailed in 74 (69%) studies, two of which reported the use of intraoperative electrophysiology. Follow-up timing was outlined by 74 (69%) studies. Mean follow-up was reported by 48 (44%) studies. Identification of the chosen time points for outcome comparison was often ambiguous. Of the prospective studies, only 41 (45%) reported follow-up rates, or the data from which it could be calculated. Many studies (19, 18%) used outcomes from ‘final follow up’ to assess their primary objectives.

### Reporting differences between study designs

Reporting differences were noted when comparing prospective with retrospective studies, and RCTs with other clinical trials (Table 1). When compared with retrospective studies, prospective studies were more likely to define the timing of follow up (p<0.01) and at which interval endpoints would be compared (p = 0.04). When compared with all other clinical trials, RCTs were more likely to define inclusion and exclusion criteria (p = 0.01) and follow up timing (p = 0.05). However they were less likely to report symptom duration (p = 0.02) or the cause of myelopathy (p<0.01) in their series. Reporting consistency did not therefore improve in all domains with higher levels of study type.

**Table 1:** Differences in reporting characteristics and study design. Significant results (p<0.05) are denoted by*.

	Prospective		Retrospective			RCT		Other Clinical Trials		
Ethics	43	47%	10	59%	0.38	9	53%	44	48%	0.73
Primary objective with time point	30	33%	3	18%	*0*.04*	4	24%	29	32%	0.84
Inclusion and exclusion criteria	63	69%	11	65%	0.71	16	94%	58	64%	*0*.01*
Cause of myelopathy specified	76	84%	11	65%	0.07	8	47%	79	87%	*<0*.01*
Comorbidities	29	32%	8	47%	0.30	5	29%	32	35%	0.9
*Comorbidity scoring system*	3	3%	2	12%	0.13	0	0%	5	5%	0.32
Symptom duration	37	41%	9	53%	0.35	3	18%	43	47%	*0*.02*
Disease level(s)	38	42%	8	47%	0.69	10	59%	36	40%	0.14
Number of treated levels	60	66%	12	71%	0.67	12	71%	60	66%	0.1
Frequency of follow up	68	75%	6	35%	*<0*.01*	15	88%	59	65%	*0*.05*

## Discussion

### Summary of findings

Heterogeneity of reported study and sample characteristics exists in DCM clinical research, even amongst studies of a higher level of evidence. This included study design characteristics, such as the requirement for ethics, clearly defined objectives and inclusion/exclusion criteria and population characteristics, such as general health status, symptom duration and disease parameters (e.g. disease level and pathology subtype). The reporting of baseline severity whilst prevalent, was reported with a variety of grading systems. This is not an unexpected finding, given its prevalence in other fields [15] and in DCM outcome reporting [3]. However it provides a challenge to effective pooled analysis [16] and interpretation of study results. As certain baseline characteristics of DCM patients are known to influence outcome[6], failure to report these entails a risk of reporting bias.

### Importance of reporting baseline characteristics

Reporting of baseline characteristics is important for the understanding of sample groups. It is also fundamental for research synthesis, as these data elements indicate to what confidence pooled outcomes can be trusted, e.g. whether patient selection was appropriate and for appraisal of methodological quality [17,18].

A key aspect of this is preventing selection bias, defined by Cochrane as the *“systematic differences between study groups*.*”* [17] Baseline data is required to make assessments of outcome bias[19,20]. The variability identified in the present study, therefore poses a limitation in DCM research, in particular:

#### 1) Incomplete recording of baseline characteristics

A recent systematic review by Tetreault et al (2015) considered prognostic factors in DCM [21]. The review identified excellent evidence suggesting that symptom duration and baseline severity are important predictors of outcomes; these factors were only reported by 46 (43%) and 97 (90%) of studies included in this review. Similarly, age may impact outcome after surgery and was reported by 107 (99%) of studies here. In addition other markers of general health status, such as diabetes, smoking and psychological factors may also influence outcomes. This highlights not only the importance of reporting such factors, but also perhaps a greater role for multivariate analysis, poorly used in the studies we reviewed.

The recording of general health status is not straightforward, as on an individual basis, diseases may be poorly represented for analysis whilst studies may focus on different co-morbidities, or use different grouping terms. Co-morbidity indexes are helpful tools to standardize this, but the bundled data may obscure the significance of key individual predictors. The significance of these indexes in DCM is not yet clearly defined.

#### 2) Incomplete recording of symptoms

The overlap of treatments for compressive cervical myelopathy and radiculopathy, in addition to their possible coexistence, has lead to their combined consideration in many studies. However, newer research indicates that the presence of radiculopathy in non-myelopathic patients with imaging evidence of cord compression is associated with higher risk of disease [22]. The impact of this on outcomes has not yet been studied, but one would expect their disease profiles to differ [23], and as such, their commonly unspecified combination is an obstacle for DCM pooled analysis. The distinction of radiculopathy from myelopathy can be difficult. This is an area in which electrophysiology could have a significant role, yet it was only specified in two studies. Of note, one of these studies identified electrophysiological markers of myelopathy severity [24] corroborated elsewhere [25].

#### 3) Incomplete recording of the pathology type

DCM is a recently proposed umbrella term to encompass cervical myelopathy due to cervical stenosis of degenerative aetiology [1]. Unification of the common clinical phenotype under a new index term will require future studies to better clarify the types of pathologies included. In addition, if the field conformed under such a term, it would lead to future, easy study identification. Whilst these ambitions are helpful, it is important not to overlook that each pathology is distinct, particularly when considered without myelopathy, and that their long-term disease profiles may differ.

### Challenges for standardisation

The development of consensus derived reporting standards has helped homogenize reporting and obviate many of the aforementioned limitations [7,26]. We intend to apply these processes to the field of DCM, to define the core outcomes and common data elements in degenerative cervical myelopathy [CODE-DCM]. The results of this systematic review, alongside further planned work, will be used to inform a DELPHI process, made up of key stakeholders including patients, care givers, professionals and industry. This project has been registered with the COMET initiative [14].

The challenge for CODE-DCM stakeholders when interpreting the findings of this systematic review will be to delineate variables present by convention or chance, from those that will make a contribution. Gender for example is almost ubiquitously reported and not known to influence outcome, whereas symptom duration is a significant predictor of outcome and was reported by less than half of studies [27]. The selection of pertinent components is key to ensure the resultant framework is concise and not an inflexible burden that could impede novel research [4].

A threat to succinct guidelines would include attempts to future proof them. As already mentioned, the ambiguity of co-morbidities is one example, but also the inclusion of promising new imaging techniques not captured in this systematic review, such as PET [28] and Diffusion Tensor Imaging [29]. Future-proofing is extremely difficult and may risk overcomplicating reporting at this time. Instead, careful consideration of future research with subsequent updates may be more appropriate [4].

### Further perspectives for DCM research

Some additional findings from this review are worth mentioning as they may represent knowledge gaps in the field of DCM. The limited use of electrophysiology and the significance of radiculomyelopathy compared with myelopathy on outcome improvements have already been mentioned. An additional area of interest is the common exclusion of patients with previous surgery. When assessing an intervention, it is understandable that potential confounders are excluded, but given this group of patients represent a significant proportion of our practice, a better understanding of their response to repeat surgery is a clinical need.

### Limitations

This series reports on the articles selected by its search strategy, which has inherent limitations addressed in our previous publication. [3]

An additional limitation distinct to the common data elements review compared to our previous core outcomes review was a greater discrepancy between authors during data extraction. This likely relates to the requirement for many elements to be interpreted from the text rather than simply copied. For example whether a study was prospective or retrospective was not always recorded, and therefore on some occasions had to be interpreted from the methodology. This risks some errors in the reporting of findings. However, the use of two authors to extract data, and the use of over 100 studies should prevent any such error impacting the overall findings. This observation would suggest a greater need for the use of reporting guidelines such as STROBE and CONSORT [19,20].

## Conclusion

Heterogeneity in the reporting of study and sample characteristics exists, even when considering higher levels of evidence. These findings echo those of outcome reporting in DCM, and further exemplify the need for the establishment of a common reporting set [3].

## Supporting information

S1 Table PRISMA Checklist for Systematic Reviews. The PRISM Checklist, including page references to the location of components in this article.
(DOC)

S2 Table Shortlisted Articles. Spreadsheet providing the initially shortlisted articles.
(XLSX)

S3 Table Included articles and Extracted Data. Spreadsheet containing the extracted data (agreed by authors BMD and MM) for all included articles.
(XLSX)
